# Reno-Metabolic Multimorbidity and Psychiatric Comorbidity: Development of a Renal–Psychiatric/Psychosomatic Burden Score in a Real-World Cohort

**DOI:** 10.3390/medicina62010066

**Published:** 2025-12-28

**Authors:** Ana Lucreția Trandafir, Oceane Colasse, Marc Cristian Ghitea, Evelin Claudia Ghitea, Timea Claudia Ghitea, Roxana Daniela Brata, Alexandru Daniel Jurca

**Affiliations:** 1 Doctoral School of Biological and Biomedical Sciences, University of Oradea, 1 University Street, 410087 Oradea, Romania; trandafir.analucretia@student.uoradea.ro; 2 Faculty of Medicine and Pharmacy, University of Oradea, 410068 Oradea, Romania; colasse.oceane@student.uoradea.ro (O.C.); ghitea.marccristian@student.uoradea.ro (M.C.G.); ghitea.evelinclaudia@student.uoradea.ro (E.C.G.); 3 Pharmacy Department, Faculty of Medicine and Pharmacy, University of Oradea, 1 University Street, 410087 Oradea, Romania; 4 Department of Medical Disciplines, Faculty of Medicine and Pharmacy, University of Oradea, 1 University Street, 410087 Oradea, Romania; alexjurca@uoradea.ro

**Keywords:** anxiety disorders, chronic kidney disease, insulin resistance, multimorbidity, psychiatric comorbidity, reno-metabolic risk, RePsy-Risk score

## Abstract

*Background and Objectives:* Renal and metabolic disorders frequently coexist with psychiatric and psychosomatic conditions, forming complex multimorbidity clusters that challenge traditional models of care. Anxiety, depression, and stress-related disorders may amplify the clinical trajectory of chronic kidney disease (CKD) and metabolic dysfunction. This study aimed to characterize the renal–psychiatric/psychosomatic burden profile of a real-world clinical cohort and to introduce a novel integrative multimorbidity score (RePsy-Risk) quantifying the combined renal, metabolic, and psychiatric burden. *Materials and Methods:* We conducted a cross-sectional analysis of 148 adult patients stratified into a reno-metabolic group (group 1) and a comparison group with other comorbidities (group 2). Clinical, biochemical, and psychiatric data were extracted from routine medical records. RePsy-Risk was constructed from three domains: renal impairment (eGFR, UACR), metabolic load (TyG index, diabetes/metabolic diagnosis), and psychiatric/psychosomatic involvement (diagnostic text-mining, psychotropic treatment). Group differences were assessed using Mann–Whitney U and *t*-tests, and associations were explored via Spearman correlation and heatmap visualization. *Results*: The reno-metabolic group exhibited significantly higher serum creatinine (1.07 vs. 0.86 mg/dL, *p* = 0.0027), a greater medication burden (7.07 vs. 5.70 drugs, *p* = 0.0007), and a higher RePsy-Risk score (mean 4.11 vs. 3.20, *p* = 0.00028). Overall, 52.0% of patients were classified as low risk, 45.3% as moderate risk, and 2.7% as high risk. RePsy-Risk correlated strongly with renal dysfunction (eGFR: ρ = –0.62; UACR: ρ = 0.38) and with metabolic load (TyG: ρ = 0.53), while psychiatric factors contributed independently (RePsy_C: ρ = 0.48). Heatmap analysis confirmed clustering of renal and metabolic domains, with psychosomatic features forming a distinct but additive dimension. *Conclusions*: Reno-metabolic disease is associated with a significantly elevated renal–psychiatric/psychosomatic burden, shaped by the interplay between impaired renal function, metabolic stress, and psychiatric comorbidity. The RePsy-Risk score offers a practical tool for capturing this multidimensional vulnerability, highlighting the need for integrated clinical strategies that simultaneously address renal, metabolic, and mental health pathways. Further validation in larger cohorts is warranted.

## 1. Introduction

Chronic kidney disease (CKD) and metabolic disorders such as type 2 diabetes, insulin resistance, dyslipidemia, and hypertension frequently coexist, forming highly prevalent and clinically challenging multimorbidity patterns. The bidirectional relationship between renal dysfunction and metabolic stress is well characterized: insulin resistance accelerates glomerular damage, while reduced kidney function contributes to systemic inflammation, oxidative stress, and impaired metabolic homeostasis. As populations age and metabolic risk factors accumulate, reno-metabolic disease has become one of the most pressing global health burdens, with implications that extend beyond traditional organ-centered care [1,2,3,4].

In parallel, psychiatric and psychosomatic disorders (including anxiety, depression, cognitive disturbances, stress-driven somatic symptoms, and sleep dysregulation) have emerged as defining components of modern multimorbidity. Described increasingly as the “disease of the century,” anxiety–depressive states exert both behavioral and biological effects that may worsen renal and metabolic trajectories. Chronic psychosomatic features were associated with dysregulation of the hypothalamic–pituitary–adrenal (HPA) axis, sympathetic overactivity, low-grade inflammation, and endothelial dysfunction, all mechanisms implicated in CKD progression and metabolic deterioration. Conversely, patients with CKD often develop psychiatric symptoms related to disease burden, altered neuroendocrine signaling, medication effects, and reduced quality of life. Thus, psychiatric involvement is not merely comorbid but physiologically interconnected with renal and metabolic disease [5,6,7]. In this manuscript, the term “psychiatric/psychosomatic burden” is used as an umbrella concept encompassing both formally diagnosed psychiatric disorders (e.g., depression, anxiety, psychotic disorders) and stress-related or psychosomatic manifestations (e.g., sleep disturbance, adjustment symptoms). The term “psychiatric” refers strictly to clinically diagnosed mental disorders or psychotropic treatment, while “psychosomatic” denotes stress-associated somatic or functional symptoms without a primary psychiatric diagnosis.

Despite the growing recognition of these interactions, multimorbidity research has historically treated renal, metabolic, and psychiatric dimensions as separate entities. Current risk stratification tools primarily focus on biochemical markers or organ-specific outcomes, rarely integrating psychological or psychosomatic components. This reductionist approach overlooks the layered nature of real-world patients, in whom biochemical impairment, metabolic dysregulation, and mental health disturbances frequently coexist and reinforce each other. There is a clear need for integrative frameworks capable of capturing the full range of biological and psychological determinants of disease severity [8,9,10,11].

In this context, the present study introduces the RePsy-Risk score, a multidimensional index combining renal impairment (eGFR, UACR), metabolic load (TyG index, metabolic diagnoses), and psychosomatic features (diagnostic text-mining and psychotropic treatment). This score was developed to address a gap in the current multimorbidity assessment landscape by quantifying renal–psychiatric/psychosomatic burden in a holistic and clinically meaningful manner. By applying the score to a real-world clinical cohort stratified into reno-metabolic versus non-reno-metabolic subgroups, the study aims to (1) characterize the multimorbidity profile associated with reno-metabolic disease, (2) assess the contribution of psychiatric factors to overall disease burden, and (3) evaluate the discriminative capacity and internal coherence of the RePsy-Risk score.

Through this integrated approach, the study provides a novel perspective on the interplay between kidney function, metabolic stress, and psychiatric morbidity, supporting the need for multidimensional diagnostic and management strategies in reno-metabolic populations. In Romania and Eastern European countries, the prevalence of chronic kidney disease, metabolic syndrome, and anxiety–depressive disorders has increased substantially over the past decade, placing a growing burden on healthcare systems. Integrated frameworks such as the International Diabetes Federation Kidney Initiative highlight the need to address renal, metabolic, and systemic complications concurrently, supporting the relevance of multidimensional risk stratification approaches in real-world cohorts.

## 2. Materials and Methods

### 2.1. Study Design and Setting

This was a cross-sectional, observational study using data extracted from a real-world clinical database including 148 patients evaluated for cardiometabolic and renal conditions. The database contained anonymized demographic, diagnostic, treatment, and laboratory information collected during routine care. The present analysis focused on the development and preliminary validation of an integrated renal–psychiatric/psychosomatic burden score (RePsy-Risk) and on the comparison between patients with reno-metabolic multimorbidity and those with other comorbidities.

### 2.2. Study Population and Cohort Classification

All 148 patients with complete information on the cohort classification variable were considered eligible. According to this variable, patients were categorized into the following groups:

- Reno-metabolic group (group 1): individuals with documented chronic kidney disease and/or reno-metabolic involvement (e.g., reduced eGFR, albuminuria, or renal diagnoses in the medical record), typically in combination with cardiometabolic disorders such as hypertension, type 2 diabetes, or dyslipidemia.
- Non-reno-metabolic group (group 2): individuals with other predominant comorbidities (e.g., purely cardiovascular, hepatic, or non-metabolic conditions), without primary classification as reno-metabolic.

Patients with missing data on all renal biomarkers (eGFR and UACR) were excluded from analyses involving the RePsy-Risk score but remained in descriptive summaries where appropriate.

### 2.3. Clinical and Laboratory Variables

For each patient, the following variables were extracted from the database:

- Demographic data: age (years).
- Renal markers: serum creatinine (mg/dL), estimated glomerular filtration rate (eGFR, mL/min/1.73 m^2^), and urinary albumin-to-creatinine ratio (UACR, mg/g).
- Metabolic markers: TyG index (triglyceride–glucose index, pre-calculated in the database), presence of type 2 diabetes mellitus and/or metabolic syndrome based on diagnostic entries.
- Psychiatric/psychosomatic variables: free-text diagnostic descriptions and the indicator variable Tratament_psihoanxiolitic (yes/no) for psychotropic or anxiolytic treatment.
- Medication burden: total number of concomitant medications recorded in the treatment list.

### 2.4. Identification of Psychiatric and Psychosomatic Comorbidity

Psychiatric and psychosomatic conditions were identified using a two-step approach:

1. Text-mining of diagnostic entries: a predefined dictionary of keywords related to mental health and psychosomatic disorders was applied to the “Diagnostic” field. The dictionary included stems such as depres, anx, panic, fobie, psiho, schizo, bipol, demen, cognitiv, insomnie, somn, adaptare, obsesiv, compulsiv.
2. Psychotropic treatment indicator: the variable Tratament_psihoanxiolitic was used to identify patients receiving antidepressants, anxiolytics, antipsychotics, mood stabilizers, or sedative–hypnotics.

Based on this information, patients were categorized into levels of psychiatric burden for the RePsy-Risk score.

This pragmatic approach was chosen to maximize feasibility in real-world clinical datasets but does not replace structured psychiatric interviews or standardized symptom severity scales. As a result, some degree of misclassification of psychiatric or psychosomatic burden is possible.

### 2.5. Development of the Renal–Psychiatric/Psychosomatic Burden Score (RePsy-Risk)

The RePsy-Risk score was developed as an exploratory, hypothesis-generating composite index intended to summarize multimorbidity burden rather than as a validated predictive model. Equal weighting of the renal, metabolic, and psychiatric/psychosomatic domains was applied to allow transparent integration of heterogeneous clinical dimensions and to avoid model overfitting in the context of a limited sample size.

Throughout the manuscript, RePsy A refers to the renal domain, RePsy B to the metabolic domain, and RePsy C to the psychiatric/psychosomatic domain.

The RePsy-Risk score was developed as an exploratory, hypothesis-generating composite index intended to summarize multimorbidity burden rather than as a validated predictive or diagnostic tool. The RePsy-Risk score was designed to integrate renal function, metabolic stress, and psychiatric/psychosomatic involvement into a single ordinal index. It comprised three domains:

#### 2.5.1. Domain A—Renal Function and Damage (0–4 Points)

Renal burden was defined using eGFR and UACR:

- eGFR ≥ 60 mL/min/1.73 m^2^ → 0 points
- eGFR 45–59 mL/min/1.73 m^2^ → 1 point
- eGFR 30–44 mL/min/1.73 m^2^ → 2 points
- eGFR < 30 mL/min/1.73 m^2^ → 3 points

An additional 1 point was added if UACR ≥ 30 mg/g (micro- or macroalbuminuria). Patients without eGFR but with available creatinine were not scored for this domain and were excluded from analyses requiring the full RePsy-Risk computation.

These eGFR and UACR thresholds were selected in accordance with KDIGO chronic kidney disease staging guidelines and are commonly applied in clinical and epidemiological renal research.

#### 2.5.2. Domain B—Reno-Metabolic Load (0–3 Points)

Metabolic burden was assessed using the TyG index and the presence of manifest metabolic disease:

- TyG < 8.5 → 0 points
- 8.5 ≤ TyG < 9.5 → 1 point
- TyG ≥ 9.5 → 2 points

An additional 1 point was assigned if type 2 diabetes mellitus and/or metabolic syndrome was documented in the diagnostic field.

TyG index thresholds were derived from previously published cardiometabolic risk stratification studies and applied here as surrogate markers of insulin resistance. These cut-offs have not been specifically validated in advanced multimorbid renal populations and are therefore used in an exploratory manner.

#### 2.5.3. Domain C—Psychiatric/Psychosomatic Domain (0–3 Points)

Psychiatric and psychosomatic involvement was graded as follows:

- 0 points: no psychiatric/psychosomatic keywords in the diagnostic text and no psychotropic/anxiolytic treatment.
- 1 point: presence of mild psychosomatic/adjustment features (e.g., sleep disturbance, adjustment disorder) in the diagnostic text, without psychotropic treatment.
- 2 points: documented psychiatric diagnosis (e.g., depression, anxiety, psychosis, bipolar disorder, dementia) or psychotropic/anxiolytic treatment marked as “Yes”.
- 3 points: ≥2 psychiatric diagnoses (e.g., depression plus anxiety; dementia plus behavioral disturbance) and psychotropic/anxiolytic treatment.

Psychiatric variables were used exclusively as components of the composite score and were not analyzed as independent outcome measures.

#### 2.5.4. Total Score and Risk Categories

The total RePsy-Risk score was calculated as the sum of Domains A, B, and C:

RePsy-Risk = A (0–4) + B (0–3) + C (0–3)

Range: 0–10 points

Patients were subsequently classified into three ordinal risk categories:

- Low renal–psychiatric/psychosomatic burden: 0–3 points
- Moderate burden: 4–6 points
- High burden: 7–10 points

The score was computed for all patients with complete data on renal, metabolic, and psychiatric domains.

### 2.6. Statistical Analysis

Statistical analyses were performed using SPSS version 30 (Armonk, NY, USA). Continuous variables were summarized as mean ± standard deviation (SD) and median with interquartile range (IQR). Categorical variables were expressed as counts and percentages.

Normality of continuous variables (e.g., creatinine, UACR, TyG index, number of medications, RePsy-Risk score) was assessed using the Shapiro–Wilk test. For comparisons between the reno-metabolic group 1 and the non-reno-metabolic group 2:

- Unpaired *t*-tests were applied to normally distributed variables.
- Mann–Whitney U tests were used for non-normally distributed variables.
- Chi-square or Fisher’s exact tests were used for categorical data (e.g., prevalence of psychiatric treatment, distribution across RePsy-Risk categories).

Spearman correlation coefficients were calculated to explore associations between the RePsy-Risk score and key biomarkers (TyG, creatinine, eGFR, UACR), as well as medication burden. Exploratory logistic or ordinal regression models may be fitted to examine whether psychosomatic features or reno-metabolic classification predicts higher RePsy-Risk categories, depending on data completeness.

A two-sided *p*-value < 0.05 was considered statistically significant. Given the exploratory nature of the study, no formal adjustment for multiple testing was applied, and results were interpreted primarily in terms of effect sizes and clinical plausibility.

Missing data were present for some laboratory and psychiatric variables. RePsy-Risk scores were calculated using a complete-case approach for patients with available data across all three domains. No imputation methods were applied due to the retrospective real-world design and heterogeneous missingness patterns. Patients with partial data were retained in descriptive analyses where applicable.

### 2.7. Ethical Considerations

The study was conducted in accordance with the Declaration of Helsinki and approved by the Institutional Ethics Committee of the University of Oradea (protocol No. 46/date 31 October 2025).

## 3. Results

### 3.1. Demographic and Baseline Characteristics of the Reno-Metabolic and Non-Reno-Metabolic Groups

A total of 141 patients met the inclusion criteria for the present analysis, of whom 81 (57.4%) were classified in the reno-metabolic group 1 and 60 (42.6%) in the non-reno-metabolic group 2. The mean age of the entire study population was 62.9 ± 14.1 years, with no clinically meaningful difference between the two groups (63.7 ± 13.8 vs. 61.8 ± 14.6 years, respectively). When stratified according to KDIGO eGFR categories, the reno-metabolic cohort included patients across CKD stages G2–G4, with the highest RePsy-Risk scores observed predominantly among individuals in stages G4–G5. This indicates that the renal domain of the score is sensitive to advanced CKD severity rather than isolated or transient renal function fluctuations.

As expected, patients in the reno-metabolic group exhibited a more pronounced renal burden. Serum creatinine levels were higher in the reno-metabolic cohort (1.07 ± 0.98 mg/dL) compared to the non-reno-metabolic group (0.86 ± 0.49 mg/dL). The distribution of albuminuria was also more unfavorable in the reno-metabolic group, with UACR values showing a broader dispersion and a greater proportion of individuals with micro- or macroalbuminuria. Median UACR was identical across groups (10.05 mg/g), but the upper quartile and extreme values were markedly higher in reno-metabolic patients, indicating more frequent renal vascular injury.

The TyG index, reflecting insulin resistance, was marginally higher in the reno-metabolic group (9.16 ± 0.72) compared with those with other comorbidities (9.05 ± 0.79), suggesting a comparable yet slightly more pronounced metabolic load in the reno-metabolic cohort. The FIB-4 index, used to estimate liver fibrosis, displayed heterogeneous values in both groups, though extreme elevations were more prevalent in the non-reno-metabolic cohort, likely driven by individuals with advanced hepatic or systemic disease.

Psychiatric and psychosomatic involvement was common across the entire population. The proportion of patients receiving psychotropic or anxiolytic therapy was similar between the groups: 22.2% in the reno-metabolic group and 23.3% in the non-reno-metabolic group. However, diagnostic-text analysis revealed qualitative differences: reno-metabolic patients more frequently presented sleep-related or adjustment-type symptoms, while classical affective disorders (depression, anxiety) were more evenly distributed. These findings support the hypothesis that kidney–metabolic dysfunction and psychosomatic manifestations coexist frequently, even when pharmacological psychiatric treatment is not uniformly prescribed.

Total medication use was substantial in both groups, consistent with multimorbidity. Patients with reno-metabolic involvement displayed a slightly higher therapeutic load (mean: 6.2 medications) compared with those in the non-reno-metabolic group (5.7 medications), reflecting the added need for nephroprotective, cardiometabolic, and antihypertensive regimens.

To clarify renal disease severity, we stratified patients by KDIGO eGFR stages. Among 143 patients with available eGFR values, 31 (21.7%) were classified as G1, 41 (28.7%) as G2, 35 (24.5%) as G3a, 28 (19.6%) as G3b, and 8 (5.6%) as G4, with no patients staged as G5. Thus, the cohort predominantly comprised mild-to-moderate CKD (G2–G3), while advanced impairment (G4) represented a small subset. This distribution helps explain the higher dispersion of serum creatinine in the reno-metabolic group, where a limited number of patients with advanced CKD likely contributed to higher creatinine outliers. Importantly, Domain A is based on KDIGO-aligned eGFR categories and albuminuria, supporting that the inverse association between RePsy-Risk and eGFR reflects graded renal severity rather than isolated fluctuations in creatinine (Table 1).

**Figure 1:**
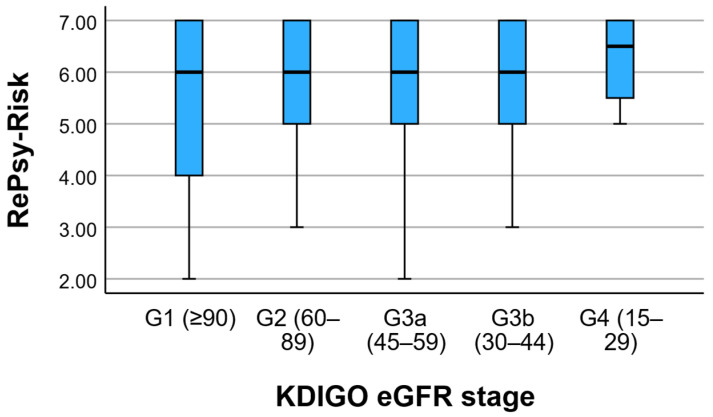
RePsy-Risk Distribution across KDIGO eGFR Stages. Boxplots show the median, interquartile range, and range of RePsy-Risk scores across KDIGO eGFR stages (G1–G4).

Albuminuria severity increased with lower eGFR stage: the proportion of A2 albuminuria rose from 9.7% in G1 to 37.5% in G4, while A3 albuminuria was rare (1.4% overall) and observed only in isolated cases. Because UACR was recorded as multiple discrete values, the initial cross-tabulation produced sparse cells (expected counts < 5 in 88.6% of cells). Therefore, we summarized UACR using KDIGO albuminuria categories (A1–A3), which is clinically standard and statistically more appropriate. KDIGO-based stratification (Table S1) showed that albuminuria severity increased with declining eGFR stage, with A2 albuminuria rising from 9.7% in G1 to 37.5% in G4. Macroalbuminuria (A3) was rare (1.4% overall) but present, consistent with the high UACR outliers observed in Figure 1. These findings indicate that mean UACR comparisons can obscure clinically relevant extremes in heterogeneous CKD populations. Figure 1 presents the distribution of RePsy-Risk scores across KDIGO eGFR stages (G1–G4).

Altogether, the demographic and baseline characteristics reveal a clinically coherent reno-metabolic cohort, distinguished primarily by higher renal burden and slightly increased metabolic strain, while psychiatric/psychosomatic comorbidity was prevalent and distributed across both groups. These patterns justify the integration of renal, metabolic, and psychiatric variables into a unified multimorbidity scoring system, such as the RePsy-Risk algorithm developed in this study.

### 3.2. Comparisons Between Reno-Metabolic and Non-Reno-Metabolic Groups

Comparative analyses between the reno-metabolic group (group 1) and the non-reno-metabolic group (group 2) identified several differences in both statistical and clinical relevance (Table 2).

Serum creatinine levels were significantly higher in the reno-metabolic group compared with the non-reno-metabolic group (1.07 vs. 0.86 mg/dL; Mann–Whitney *p* = 0.0027), supporting the internal validity of the cohort classification and reflecting the expected renal impairment in patients with reno-metabolic disease. Although reno-metabolic patients showed higher median values and more extreme measurements of UACR, the mean UACR did not differ significantly between groups (32.1 vs. 23.5 mg/g; *p* = 0.7348), consistent with the substantial inter-individual variability typically observed for albuminuria.

The TyG index was slightly higher in the reno-metabolic group (9.16 vs. 9.05), but this difference was not statistically significant (Mann–Whitney *p* = 0.1126), indicating a broadly comparable degree of insulin resistance between groups. This likely reflects the high prevalence of metabolic syndrome across the entire study population. Similarly, FIB-4 values, used as a surrogate marker of liver fibrosis, did not differ significantly between groups (*p* = 0.3915), with both cohorts exhibiting heterogeneous distributions and occasional outliers.

In contrast, the overall medication burden was significantly greater in the reno-metabolic group than in the non-reno-metabolic group (7.07 vs. 5.70 medications; *t*-test *p* = 0.0007). This finding highlights the higher degree of multimorbidity in reno-metabolic patients, who commonly require multiple therapeutic classes, including antihypertensive, antidiabetic, lipid-lowering, and nephroprotective agents.

Age did not differ significantly between the two groups (66.7 vs. 65.4 years; *p* = 0.3467), suggesting that the observed differences in renal and therapeutic parameters were not driven by age alone.

**Figure 2:**
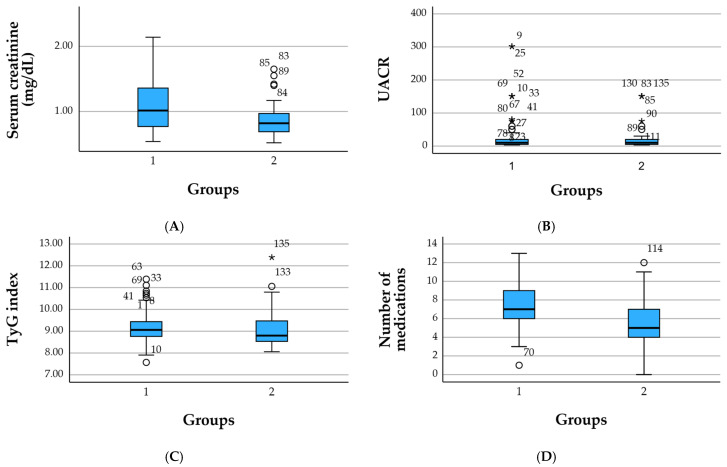
Boxplots of key renal, metabolic, and therapeutic parameters by clinical group. (**A**) Serum creatinine (mg/dL), (**B**) urinary albumin-to-creatinine ratio (UACR, mg/g), (**C**) TyG index, and (**D**) number of concomitant medications are shown for the reno-metabolic group (group 1) and the non-reno-metabolic group (group 2). Boxes represent the interquartile range (IQR), horizontal lines indicate medians, whiskers depict 1.5 × IQR, and circles denote outliers. The figure illustrates higher creatinine levels and a greater medication burden in reno-metabolic patients, with broadly similar TyG values across groups. Circles (○) denote mild outliers and the asterisk (*) indicates an extreme outlier.

Overall, patients in the reno-metabolic group were characterized by poorer renal function and a higher treatment burden, whereas metabolic markers such as TyG and FIB-4 showed similar distributions across groups. These findings indicate that the reno-metabolic cohort is clinically distinguished primarily by renal impairment and polypharmacy rather than by age or global metabolic differences, supporting the rationale for integrating renal, metabolic, and psychiatric dimensions into a unified multimorbidity index such as the RePsy-Risk score (Figure 2).

### 3.3. Distribution of the Renal–Psychiatric/Psychosomatic Burden Score (RePsy-Risk)

The RePsy-Risk score was successfully computed for all 148 patients based on available renal, metabolic, and psychiatric/psychosomatic indicators. The distribution of the total score revealed three distinct risk strata: Low, Moderate, and High burden (Figure 2). The small number of patients classified in the high-risk category limits statistical inference regarding this subgroup and warrants cautious interpretation.

Overall, 77 patients (52.0%) were classified as having a Low RePsy-Risk, 67 patients (45.3%) fell into the Moderate risk category, while only 4 individuals (2.7%) exhibited a High renal–psychiatric/psychosomatic burden. This distribution reflects a population-wide predominance of mild-to-moderate multimorbidity, with a small but clinically relevant subset presenting substantial combined renal, metabolic, and psychosomatic features.

Patients in the Moderate and High categories accumulated points predominantly from two domains:

Renal domain (A):

Albuminuria ≥ 30 mg/g and reduced eGFR were the primary drivers of upward score shifts, especially among reno-metabolic individuals.

Psychiatric/psychosomatic domain (C):

The presence of ≥2 psychiatric keywords (e.g., depression + anxiety) combined with active psychotropic treatment contributed meaningfully to high scores.

The metabolic domain (B) showed a more homogeneous distribution across categories, consistent with the overall high TyG index observed in the cohort, reflecting pervasive insulin resistance.

The High-risk group, though numerically small, is clinically important: all four individuals had advanced renal involvement (eGFR < 45 mL/min/1.73 m^2^ and/or macroalbuminuria), significant metabolic load (TyG ≥ 9.5), and documented psychiatric comorbidity with psychotropic treatment. This triad suggests a particularly vulnerable multimorbidity phenotype, in line with the conceptual purpose of the RePsy-Risk score.

**Figure 3:**
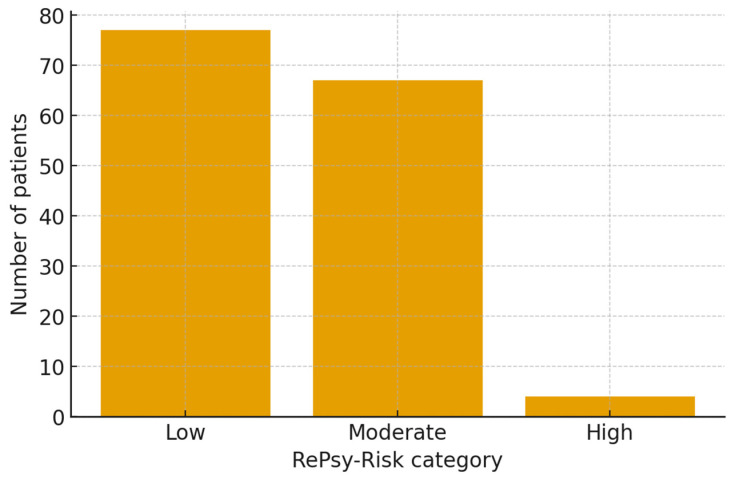
Distribution of the renal–psychiatric/psychosomatic burden score (RePsy-Risk) in the reno-metabolic (group 1) and non-reno-metabolic (group 2) cohorts. Patients were classified into three ordinal risk categories (Low (0–3 points), Moderate (4–6 points), and High (7–10 points)) according to the composite RePsy-Risk score. Bars represent the proportion of patients in each category within the two groups, illustrating the shift towards higher renal–psychiatric/psychosomatic burden among reno-metabolic individuals.

These findings support the discriminative capacity of the scoring algorithm and justify its use in subsequent analyses examining the association between reno-metabolic burden, psychiatric comorbidity, and global multimorbidity profiles (Figure 3).

### 3.4. Correlations Between the RePsy-Risk Score and Renal, Metabolic, Psychiatric, and Treatment Variables

Spearman correlation analysis identified several clinically relevant associations between the RePsy-Risk score and its component biomarkers (Table 3). As expected, the total RePsy-Risk score was strongly correlated with both the renal domain (RePsy A; ρ = 0.76) and the metabolic domain (RePsy B; ρ = 0.63), indicating that the scoring system effectively reflects cumulative multimorbidity across these dimensions.

Renal impairment emerged as a key contributor to the overall score. The RePsy-Risk score showed a moderate inverse correlation with eGFR (ρ = –0.62), demonstrating that declining renal function is associated with a higher global burden. In addition, UACR, a marker of renal microvascular injury, was positively correlated with the total score (ρ = 0.38) and showed an even stronger association with the renal domain itself (RePsy A; ρ = 0.51). These patterns are consistent with the higher prevalence of albuminuria and reduced filtration capacity observed in the reno-metabolic cohort.

The TyG index, used as a surrogate marker of insulin resistance, was moderately correlated with the total RePsy-Risk score (ρ = 0.53). Its strongest association was with the metabolic domain (RePsy B; ρ = 0.83), supporting the internal coherence and construct validity of this component. Together, these findings underscore the contribution of metabolic stress to the broader renal–psychiatric/psychosomatic burden multimorbidity framework.

The psychiatric/psychosomatic domain (RePsy C) also showed a moderate correlation with the total RePsy-Risk score (ρ = 0.48), indicating that psychosomatic burden contributes meaningfully to the overall risk profile. However, correlations between this domain and individual biochemical markers (eGFR, UACR, creatinine, TyG) were minimal, suggesting that psychosomatic features represent an independent dimension of multimorbidity rather than a direct reflection of biochemical disease severity. This supports the conceptualization of psychiatric comorbidity as a parallel axis of vulnerability, rather than a simple downstream consequence of renal or metabolic dysfunction.

The total number of medications demonstrated a weak-to-moderate positive correlation with the RePsy-Risk score (ρ = 0.19), consistent with increasing treatment complexity in patients with greater multimorbidity. Notably, correlations between medication burden and TyG (ρ = 0.28) were stronger than those with renal markers, suggesting that polypharmacy in this cohort is more closely linked to metabolic disease management than to renal impairment alone.

Overall, the correlation matrix confirms that the RePsy-Risk score captures a multidimensional pattern of multimorbidity, driven primarily by renal dysfunction and metabolic stress, with psychosomatic burden contributing an additional and largely independent dimension. The balanced distribution of correlations across domains supports the integrative design and conceptual coherence of the scoring system.

### 3.5. Heatmap Visualization of Correlations Between RePsy-Risk and Clinical Biomarkers

**Figure 4:**
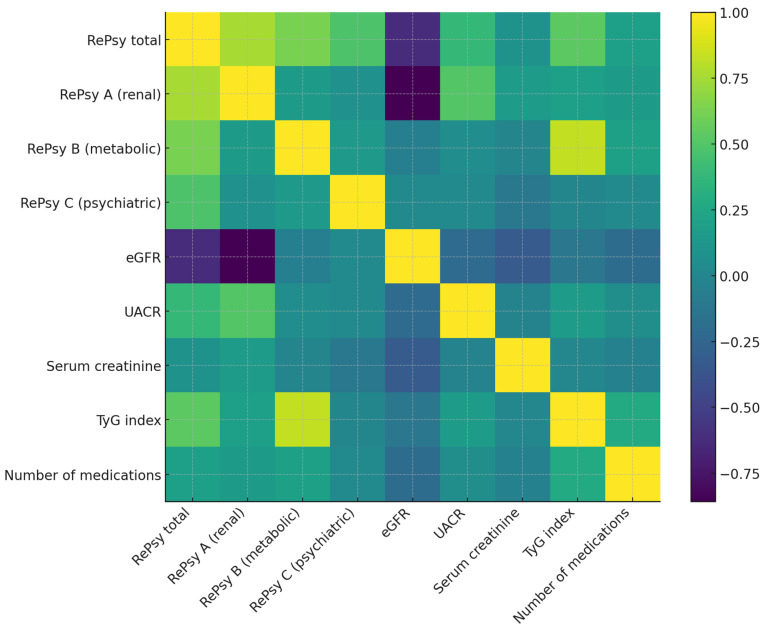
Spearman correlation heatmap between RePsy-Risk and clinical variables. Positive correlations are shown in lighter shades and negative correlations in darker shades. This heatmap illustrates the Spearman correlation coefficients between the components of the RePsy-Risk score (RePsy total, RePsy A—renal domain, RePsy B—metabolic domain, RePsy C—psychiatric domain) and key clinical biomarkers, including estimated glomerular filtration rate (eGFR), urinary albumin-to-creatinine ratio (UACR), serum creatinine, TyG index, and the total number of medications. Positive correlations are shown in lighter colors, and negative correlations in darker colors. The strongest correlations observed include a marked inverse relationship between RePsy A (renal domain) and eGFR and positive associations between RePsy B (metabolic domain) and the TyG index, consistent with the intended structure of the score. Color intensity reflects correlation strength (|ρ|): weak (<0.3), moderate (0.3–0.6), and strong (>0.6). A color bar indicates the full correlation range (ρ from −1 to +1).

The heatmap displays Spearman correlation coefficients between the RePsy-Risk components (RePsy total, RePsy A—renal domain, RePsy B—metabolic domain, RePsy C—psychiatric domain) and key clinical biomarkers, including eGFR, UACR, serum creatinine, TyG index, and number of medications. Warmer colors represent positive correlations, while cooler colors indicate negative correlations. The diagonal line reflects perfect correlations (ρ = 1.0). The matrix illustrates the strong inverse relationship between the renal domain score (RePsy A) and eGFR, as well as the positive associations of the metabolic domain (RePsy B) with the TyG index (Figure 4).

### 3.6. Comparative Analysis of the RePsy-Risk Score Between Reno-Metabolic and Non-Reno-Metabolic Groups

The RePsy-Risk score was compared between patients classified as reno-metabolic (group 1) and those with other comorbidities (group 2). Significant differences were observed across the groups, confirming that the reno-metabolic cohort carries a substantially higher renal–psychiatric/psychosomatic burden.

Patients in the reno-metabolic group exhibited a higher mean RePsy-Risk score compared with those in the non-reno-metabolic group (4.11 vs. 3.20, respectively). Median values followed a similar pattern (4 vs. 3). Distributional testing showed non-normality in one or both groups (Shapiro–Wilk *p* < 0.05), prompting the use of the Mann–Whitney U test. The difference between groups was statistically significant (*p* = 0.00028), indicating a meaningful divergence in multimorbidity profiles.

When examining score components, renal impairment (RePsy A) accounted for most of the between-group difference, consistent with elevated creatinine levels and a higher prevalence of albuminuria among reno-metabolic patients. However, metabolic load (RePsy B) and psychiatric burden (RePsy C) also contributed to the total score, indicating that the reno-metabolic group accumulates multimorbidity across multiple axes, not only through renal parameters.

In the reno-metabolic cohort, 4 points represented the central tendency (median), corresponding to patients with moderately impaired renal function, elevated TyG index, and mild-to-moderate psychiatric involvement. By contrast, the non-reno-metabolic group predominantly clustered in the low-burden range, reflecting preserved renal function and lower cumulative metabolic–psychosomatic features.

These results validate the discriminative ability of the RePsy-Risk score in distinguishing multimorbidity severity between clinically defined subgroups and reinforce the conceptual interdependence of renal, metabolic, and psychiatric components in reno-metabolic disease (Table 4).

## 4. Discussion

This study explored the multidimensional interplay between renal impairment, metabolic dysfunction, and psychiatric or psychosomatic involvement using a newly developed composite index, the RePsy-Risk score, to quantify global multimorbidity in a real-world clinical population. Our findings indicate that patients with reno-metabolic disease exhibit a significantly higher combined renal and psychosomatic burden compared with individuals presenting other comorbidity profiles. These differences were reflected both in renal biomarkers and in cumulative RePsy-Risk scores. Importantly, the results also underscore the independent contribution of psychiatric factors within the multimorbidity framework, supporting the view that anxiety, depression, and related conditions constitute a central dimension of contemporary disease vulnerability. Equal weighting of score domains was chosen for exploratory integration and transparency and does not imply equivalent clinical severity across domains. In practice, advanced renal dysfunction contributes disproportionately to high RePsy-Risk values, while psychiatric burden adds an independent but non-dominant dimension. Future studies should explore weighted or outcome-calibrated versions of the score to better reflect differential clinical impact.

A key observation was the markedly higher RePsy-Risk score observed in the reno-metabolic cohort, which was primarily associated with reduced eGFR and increased albuminuria—two established indicators of chronic kidney disease (CKD) severity. This finding is consistent with extensive evidence showing that CKD commonly coexists with cardiometabolic disorders such as hypertension, diabetes, and dyslipidemia, forming a pathophysiological continuum characterized by endothelial dysfunction, chronic inflammation, and microvascular injury. The moderate-to-strong correlations between the RePsy-Risk score and renal markers (eGFR and UACR) further support the construct validity of the renal domain within the scoring system [1,12,13,14,15,16].

Metabolic dysregulation, as reflected by the TyG index, also made a substantial contribution to the overall RePsy-Risk score. This observation aligns with prior research linking insulin resistance to both progressive renal dysfunction and increased cardiovascular risk. The strong association between TyG and the metabolic domain of the RePsy-Risk algorithm highlights its relevance as a core component of reno-metabolic multimorbidity. In the context of the rising global prevalence of metabolic syndrome and its close association with early and subclinical CKD, the TyG index emerges as a useful cross-domain biomarker that integrates glycemic and lipid-related metabolic pathways [16,17,18,19,20,21,22,23,24].

A central novelty of this study lies in the integration of psychiatric and psychosomatic factors into the multimorbidity analysis. The psychiatric domain correlated moderately with the total RePsy-Risk score but displayed weak associations with biochemical markers, indicating that psychosomatic features constitute an independent and additive dimension, not merely a downstream consequence of metabolic or renal dysfunction. Residual conceptual overlap between score components cannot be entirely excluded. This is clinically meaningful: anxiety, depression, sleep disturbances, and cognitive strain may amplify disease burden through behavioral pathways (e.g., reduced treatment adherence), biological mechanisms (e.g., autonomic dysregulation, chronic inflammation), or medication effects (e.g., psychotropics and metabolic dysregulation) [25,26,27,28].

Given the cross-sectional design and the limited size of the high-risk stratum, the RePsy-Risk score should be interpreted as a descriptive and stratification tool rather than a prognostic instrument. Longitudinal studies are required to determine whether higher RePsy-Risk categories are associated with adverse renal, metabolic, cardiovascular, or psychiatric outcomes over time.

These findings resonate with a broader clinical and societal trend, where anxiety and depressive disorders have become defining elements of “the disease of the century.” Their high prevalence across both reno-metabolic and non-reno-metabolic patients in this study underscores the pervasive interaction between mental stress and chronic somatic illness. The inclusion of a psychiatric component within the RePsy-Risk score therefore reflects an important step toward a holistic multimorbidity model, capturing dimensions of illness that are often overlooked in traditional biomedical indices.

Although the reno-metabolic group had a higher medication count, the weak correlation with RePsy-Risk likely reflects that medication number captures treatment complexity rather than biological burden. In real-world reno-metabolic care, polypharmacy frequently includes guideline-directed nephroprotective and cardiometabolic therapies, which may increase medication count without directly mirroring renal injury severity. Detailed medication-class analysis (including potentially nephrotoxic exposures) was not feasible in the present dataset and represents an important direction for future work.

Another notable finding is that therapeutic burden (number of medications) correlated modestly with RePsy-Risk, particularly with the metabolic domain. This suggests that polypharmacy appears to be more closely related to cardiometabolic treatment needs than to renal or psychiatric conditions alone. Nonetheless, the increased medication load in reno-metabolic individuals reflects the clinical complexity of managing such patients and reinforces the need for integrative pharmacological strategies aimed at reducing interactions, simplifying regimens, and improving adherence [29,30,31].

Taken together, this study demonstrates that renal, metabolic, and psychiatric components form an interconnected triad of multimorbidity, with each dimension adding unique explanatory value to the overall burden. The RePsy-Risk score successfully differentiates patients with reno-metabolic disease from those with other comorbidities and shows strong internal coherence across domains. By capturing multidimensional vulnerability in a single metric, RePsy-Risk may serve as a useful stratification tool for clinicians assessing patients with complex multimorbidity profiles. The weak correlation between medication count and RePsy-Risk likely reflects the fact that polypharmacy in this cohort predominantly represents guideline-directed cardiometabolic and nephroprotective therapy rather than disease severity per se. As such, medication number captures treatment complexity rather than biological burden, explaining its modest association with the composite score. Detailed drug class stratification was beyond the scope of the present analysis and warrants dedicated investigation in future studies.

Future work should focus on external validation of the scoring system in larger cohorts, assessment of its prognostic value for renal, cardiovascular, or psychiatric outcomes, and exploration of its utility in integrated care pathways. Additionally, refining the psychiatric domain to incorporate symptom severity scales or structured diagnostic data may enhance precision and facilitate broader applicability. These findings should be interpreted within the context of a single-center real-world cohort and may not be directly generalizable to other healthcare systems or populations with differing diagnostic and treatment practices. Future studies should evaluate alternative weighting strategies using data-driven approaches such as factor analysis, regression-based weighting, or outcome-based calibration.

### Strengths and Limitations

This study presents several important strengths. First, it introduces and operationalizes a novel, integrative multimorbidity index (the RePsy-Risk score) designed to capture the combined renal, metabolic, and psychiatric burden within a real-world clinical cohort. By incorporating three biologically and clinically distinct domains, the score reflects the complexity of renal–psychosomatic interactions more comprehensively than single-dimension markers. External validation in independent cohorts is essential before clinical implementation. The use of objective biomarkers (eGFR, UACR, TyG index) alongside psychiatric/psychosomatic features and therapeutic data enhances the ecological validity of the model. Additionally, the study leverages a real-world dataset, allowing the findings to reflect the heterogeneity and multimorbidity patterns commonly encountered in clinical practice rather than the artificially controlled distributions seen in randomized trials. The application of text-mining for psychiatric identification represents a pragmatic and replicable method for extracting mental health information from unstructured clinical records. Exclusion of incomplete cases for composite score calculation may introduce selection bias. Confidence intervals were not calculated for all exploratory analyses, which may limit precision estimates.

The identification of psychiatric and psychosomatic involvement relied on keyword-based diagnostic text mining and treatment records rather than standardized psychiatric assessments, which may have led to under- or overestimation of psychological burden in some patients.

However, several limitations must be acknowledged. The cross-sectional design precludes causal inference, limiting the ability to determine temporal relationships between renal decline, metabolic dysfunction, and psychiatric comorbidity. Sample size constraints, especially within the high-risk RePsy category, reduce the precision of comparative estimates and may limit generalizability. Some variables, particularly eGFR, UACR, and psychiatric descriptors, contained missing or unstructured data, which may introduce classification bias in the scoring algorithm. The identification of psychosomatic features relied primarily on keyword-based text-mining and medication records rather than structured psychiatric assessments, which may underestimate or overestimate psychological morbidity. Furthermore, although RePsy-Risk demonstrates internal consistency, it requires external validation in larger, diverse populations and should be tested for predictive value regarding clinical outcomes such as CKD progression, hospitalization, or mental health deterioration. External validation in an independent cohort was not performed and is necessary before clinical implementation of the RePsy-Risk score. Future studies should validate the score across different healthcare settings and populations and evaluate its prognostic utility using longitudinal outcomes.

Although mean UACR values did not differ significantly between groups, inspection of individual values revealed that macroalbuminuria clustered predominantly among patients with advanced CKD stages, underscoring the limitations of mean-based comparisons in heterogeneous renal populations.

Biomarker cut-offs were extrapolated from guideline-based and population-level studies and may not optimally reflect risk thresholds in complex multimorbid renal patients.

Key confounders such as socioeconomic status, CKD duration and severity, inflammatory markers, lifestyle factors, and cardiovascular comorbidity were not available for adjustment. Therefore, observed associations should be considered unadjusted and exploratory.

The equal weighting of score components was chosen for pragmatic integration and does not imply equivalent clinical impact.

Despite these limitations, the study provides a valuable framework for understanding the interplay between renal, metabolic, and psychiatric dimensions of multimorbidity and lays the groundwork for future longitudinal and interventional research.

## 5. Conclusions

This study demonstrates that reno-metabolic disease is closely intertwined with psychosomatic features, forming a multidimensional multimorbidity cluster that extends beyond classical biochemical markers. At its current stage of development, the RePsy-Risk score serves as both a descriptive tool and a stratification tool that captures multidimensional multimorbidity in real-world reno-metabolic populations, providing a foundation for future validation and prognostic research. Using the novel RePsy-Risk score, we identified significantly higher renal–psychiatric/psychosomatic burden in patients with reno-metabolic comorbidities compared with those exhibiting other chronic conditions. The score effectively integrated renal dysfunction, metabolic stress, and psychiatric involvement, capturing a clinically coherent and discriminative pattern of vulnerability. Importantly, psychiatric and psychosomatic factors contributed independently to the total burden, underscoring their role as parallel determinants of health rather than mere consequences of somatic disease. These findings highlight the need for care models that address renal, metabolic, and mental health domains simultaneously. Future research should validate the RePsy-Risk score in larger and longitudinal cohorts, explore its prognostic utility, and assess whether integrated interventions targeting both metabolic and mental health pathways can mitigate global multimorbidity in reno-metabolic populations.

## Figures and Tables

**Table 1:** Distribution of KDIGO eGFR stages (*n* = 143 with available eGFR).

KDIGO eGFR Stage	Definition (mL/min/1.73 m^2^)	*n*	%
G1	≥90	31	21.7
G2	60–89	41	28.7
G3a	45–59	35	24.5
G3b	30–44	28	19.6
G4	15–29	8	5.6
G5	<15	0	0.0
Total		143	100.0

KDIGO staging was derived from eGFR values available in the database; 5 patients had missing eGFR and could not be staged.

**Table 2:** Comparison of key variables between reno-metabolic (group 1) and non-reno-metabolic (group 2) groups.

Variable	Mean (Group 1)	Mean (Group 2)	Statistical Test	*p*-Value
Serum creatinine (mg/dL)	1.0736	0.8622	Mann–Whitney	0.0027
UACR (mg/g)	32.09	23.47	Mann–Whitney	0.7348
TyG index	9.163	9.049	Mann–Whitney	0.1126
FIB-4 score	1.164	1.461	Mann–Whitney	0.3915
Number of medications	7.07	5.70	*t*-test	0.0007
Age (years)	66.74	65.40	Mann–Whitney	0.3467

UACR = urinary albumin-to-creatinine ratio, TyG index = triglyceride–glucose index, FIB-4 = Fibrosis-4 score, mg/dL = milligrams per deciliter, mg/g = milligrams per gram. Medication count reflects the number of active medications documented at assessment; drug classes were not available for subclassification.

**Table 3:** Spearman correlation coefficients among RePsy-Risk components and clinical biomarkers.

Variable	RePsy Total	RePsy A	RePsy B	RePsy C
eGFR	–0.62	–0.86	–0.05	0.02
UACR	0.38	0.51	0.06	0.03
Creatinine	0.09	0.17	–0.01	–0.11
TyG index	0.53	0.19	0.83	–0.00
Number of medications	0.19	0.15	0.20	0.03

eGFR = estimated glomerular filtration rate; UACR = urinary albumin-to-creatinine ratio; TyG = triglyceride–glucose index; RePsy total = total RePsy score; RePsy A = renal domain; RePsy B = metabolic domain; RePsy C = psychiatric/psychosomatic domain.

**Table 4:** Comparison of RePsy-Risk between reno-metabolic (group 1) and non-reno-metabolic (group 2) groups.

Parameter	Reno-Metabolic (Group 1)	Other Comorbidities (Group 2)	*p*-Value	Test
RePsy-Risk total score (mean ± SD)	4.11 ± 1.19	3.20 ± 1.53	0.00028	Mann–Whitney U

SD = standard deviation, *p*-value = statistical significance.

## Data Availability

The data presented in this study are available on request from the corresponding author. The data are not publicly available due to privacy or ethical restrictions.

## Supplementary Materials

The following supporting information can be downloaded at: https://www.mdpi.com/article/10.3390/medicina62010066/s1, Table S1: Distribution of KDIGO eGFR stages and KDIGO albuminuria categories (UACR) (n = 143 with eGFR available).

## Abbreviations

The following abbreviations are used in this manuscript:

ALT	Alanine aminotransferase
AST	Aspartate aminotransferase
CKD	Chronic kidney disease
eGFR	Estimated glomerular filtration rate
FIB-4	Fibrosis-4 score
HPA axis	Hypothalamic–pituitary–adrenal axis
IQR	Interquartile range
RePsy-Risk	Renal–psychiatric/psychosomatic burden score
RePsy A	Renal domain of the RePsy-Risk score
RePsy B	Metabolic domain of the RePsy-Risk score
RePsy C	Psychiatric/Psychosomatic domain of the RePsy-Risk score
SD	Standard deviation
SPSS	Statistical Package for the Social Sciences
TyG index	Triglyceride–glucose index
UACR	Urinary albumin-to-creatinine ratio
mg/dL	Milligrams per deciliter
mg/g	Milligrams per gram
