# Supplementary material for: Reno-Metabolic Multimorbidity and Psychiatric Comorbidity: Development of a Renal–Psychiatric/Psychosomatic Burden Score in a Real-World Cohort
Source: Medicina (Kaunas). 2025 Dec 28;62(1):66. doi: 10.3390/medicina62010066 (PMC12842813; doi:10.3390/medicina62010066)
Supplement: Supplementary file 1 [file medicina-62-00066-s001.zip › medicina-4041488-supplementary.pdf]

Supplementary Table S1

Table S1. Distribution of KDIGO eGFR stages and KDIGO albuminuria categories (UACR) (n = 143 with eGFR available).

KDIGO eGFR stage	n (%)	A1 (<30) n (%)	A2 (30–300) n (%)	A3 (>300) n (%)	RePsy-Risk Mean (SD)
G1 (≥90)	31 (21.7)	28 (90.3)	3 (9.7)	0 (0.0)	5.3 (1.7)
G2 (60–89)	41 (28.7)	33 (80.5)	7 (17.1)	1 (2.4)	5.8 (1.2)
G3a (45–59)	35 (24.5)	27 (77.1)	7 (20.0)	1 (2.9)	5.6 (1.2)
G3b (30–44)	28 (19.6)	19 (67.9)	9 (32.1)	0 (0.0)	5.9 (1.2)
G4 (15–29)	8 (5.6)	5 (62.5)	3 (37.5)	0 (0.0)	6.2 (0.8)
Total	143 (100.0)	112 (78.3)	29 (20.3)	2 (1.4)	5.7 (1.2)

KDIGO eGFR stages were defined as G1 (≥90), G2 (60–89), G3a (45–59), G3b (30–44), G4 (15–29), and G5 (<15) mL/min/1.73 m^2. Albuminuria categories were defined as A1 (<30), A2 (30–300), and A3 (>300) mg/g. No G5 cases were observed among patients with available eGFR.
